# Surgical Treatment of Early-Onset Scoliosis: Traditional Growing Rod vs. Magnetically Controlled Growing Rod vs. Vertical Expandable Prosthesis Titanium Ribs

**DOI:** 10.3390/jcm14010177

**Published:** 2024-12-31

**Authors:** Bruna Maccaferri, Francesco Vommaro, Chiara Cini, Giuseppe Filardo, Luca Boriani, Alessandro Gasbarrini

**Affiliations:** 1 Spine Surgery Unit, IRCSS Istituto Ortopedico Rizzoli, 40136 Bologna, Italy; bruna.maccaferri@ior.it (B.M.); francesco.vommaro@ior.it (F.V.); luca.boriani@ior.it (L.B.); alessandro.gasbarrini@ior.it (A.G.); 2 Faculty of Biomedical Sciences, Università della Svizzera Italiana, 6962 Lugano, Switzerland; ortho@gfilardo.com; 3 Department of Biomedical and Neuromotor Sciences, University of Bologna, 40127 Bologna, Italy

**Keywords:** early-onset scoliosis, traditional growing rod, magnetically controlled growing rod, vertical expandable prosthesis titanium ribs

## Abstract

**Objectives:** Severe early-onset scoliosis (EOS) can be addressed by different growth-friendly approaches, although the indications of each technique remain controversial. The aim of this study was to compare, in a large series of patients, the potential and limitations of the different distraction-based surgical techniques to establish the most suitable surgical approach to treat EOS. **Methods:** We conducted a retrospective observational cohort study evaluating 62 EOS cases treated between January 2002 and December 2021 with a traditional growing rod (TGR), a magnetically controlled growing rod (MCGR) and vertical expandable prosthesis titanium ribs (VEPTR) at IRCSS Istituto Ortopedico Rizzoli, Bologna, Italy. The patients included had a mean age of 7 years and a mean follow-up of 36 months. The COBB angle was measured on x-rays at preoperative, early postoperative, and end of follow-up, and complications were recorded. **Results:** in our cohort, VEPTR was mainly used in congenital scoliosis (50% vs. a mean value of 25.8%) and syndromic scoliosis (42.9% vs. a mean value of 25.8%). MCGR was mainly used in idiopathic scoliosis (73.9% vs. an average value of 41.9%). TGR was mostly used in muscular neurology EOS (16% vs. an average value of 6.5%). The collected data show a similar deformity correction rate in growing-rod implants in VEPTR, TGR, and MCGR. The mean curve reduction was 25.8 95% CI (21.8–29.8) (*p* < 0.0005). Compared with preoperative measurements, significant differences in curve magnitude correction between subgroups occurred at the final treatment measurements, when patients with MCGR had a significantly larger correction (53.2° ± 20.84 in %33.9 con DS ± 14.27) than VEPTR (27.12°± 19.13 in %19.7° ± 13.7). **Conclusions:** Different growing-rod techniques are applied based on EOS etiology. While all EOS etiologies benefited from this surgical approach, congenital EOS had poorer results. Overall, MCGR has been the preferred option for idiopathic EOS and appears to be the most effective in correcting the primary curve.

## 1. Introduction

Early-onset scoliosis (EOS) refers to a spinal deformity that occurs before the age of 10 years old according to different etiologies: idiopathic, congenital, neuromuscular, and syndromic [1]. EOS has a potentially poor prognosis, with curve progression increasing the morbidity and mortality risk [2,3,4]. For patients who do not respond to conservative management or present at an advanced stage, surgical intervention is indicated [5]. Unfortunately, severe EOS management represents a challenge for spine surgeons due to the young age of these patients and their growing spine. Performing conventional spinal fusion early in children may result in thoracic insufficiency syndrome, growth retardation, cosmetic issues [2,6], and even respiratory failure and risk of mortality [7,8,9]. This is further aggravated by common complex medical comorbidities presented by these patients, with operations presenting morbidity and mortality risk [5,10,11,12]. Thus, more attention has been recently given to allowing the development of a functional pulmonary system through spine and chest wall growth-sparing techniques.

The purpose of growth-friendly techniques is achieving and maintaining deformity correction during the spine growth while the lungs develop adequately. There are a variety of growth-friendly techniques, generally categorized into three types according to the amount of correction force applied: distraction-based, compression-based, and growth-guided. The distraction-based systems are the most commonly used approach, relying on a mechanical distractive force exerted on the spine segments, ribs, and/or pelvis. It includes traditional growing rods (TGR), magnetically controlled growing rods (MCGR), and vertical expandable prosthesis titanium ribs (VEPTR) [5]. TGRs are the most frequently used growth-friendly technique and have been effective in managing EOS, although they requires repeated lengthening surgical procedures every 6 months, resulting in high complication rates, high costs due to multiple planned and unplanned surgical procedures, and a high psychosocial burden on the individual. MCGRs and VEPTRs have been introduced to overcome the need for repeated surgical procedures, with promising results [13]. However, the indication on the most suitable surgical approach to address EOS remains controversial.

The aim of this study was to compare, in a large series of patients, the potential and limitations of the different distraction-based techniques to establish the most suitable surgical approach for patients affected by EOS.

## 2. Materials and Methods

### 2.1. Study Design and Selection Criteria

An observational cohort study was performed, retrospectively evaluating medical records of EOS patients treated between January 2002 and December 2021 at IRCSS Istituto Ortopedico Rizzoli, Bologna, Italy, a national referral center. Based on the classification C-EOS, EOS etiology in these patients was classified as idiopathic, congenital, neuromuscular, and syndromic [14]. The inclusion criteria comprised patients diagnosed with EOS aged ≤ 10 years old at the time of the first surgery; surgical treatment with TGR, MCGR, VEPTR techniques; no history of previous surgery; radiographic Cobb angle greater than 30°; and at least 24 months of outpatient follow-up. The patients were excluded if diagnosed with EOS treated conservatively; aged > 10 years at the index surgery; not reaching a post-operative follow-up of minimum 2 years; no consensus given; or previous history of spinal surgery. The latest follow-up was defined as the most recent visit before a definitive spinal fusion.

### 2.2. Outcomes of Interest

The medical records, including outpatient, inpatient, and operative notes, were accurately reviewed to obtain demographic information (gender, EOS etiology, age at first operation, type of surgical technique, clinical follow-up information, number of lengthening procedures, average number of months between the lengthening procedures) for each patient.

The following radiographic parameters were assessed: major coronal curve correction (Cobb angle), T1–T12 kyphosis, change in spine height T1–S1, and thoracic high T1–T12 [15]. Among these, we evaluated the correction of the major coronal curve resulting from the different surgical approaches. Measurements on X-rays were performed at preoperative, early postoperative, and final follow-up by two independent observers in consensus.

Complications for each patient and the rate of revision surgeries were recorded as well. Complications were divided into two groups: device-related and disease-related. Infections of the surgical site (SSI), neurological complications, and implant issues (rod fracture and anchor failure) were considered complications associated with the device itself and were categorized as device-related complications. Event-related complications (i.e., minor wound infection, surgical infection), deterioration of spinal balance (including junctional kyphosis), pneumonia, and sepsis associated with repeated surgeries were classified as disease-related complications.

### 2.3. Surgical Techniques

#### 2.3.1. TGR: Traditional Growing Rods

**Figure 1:**
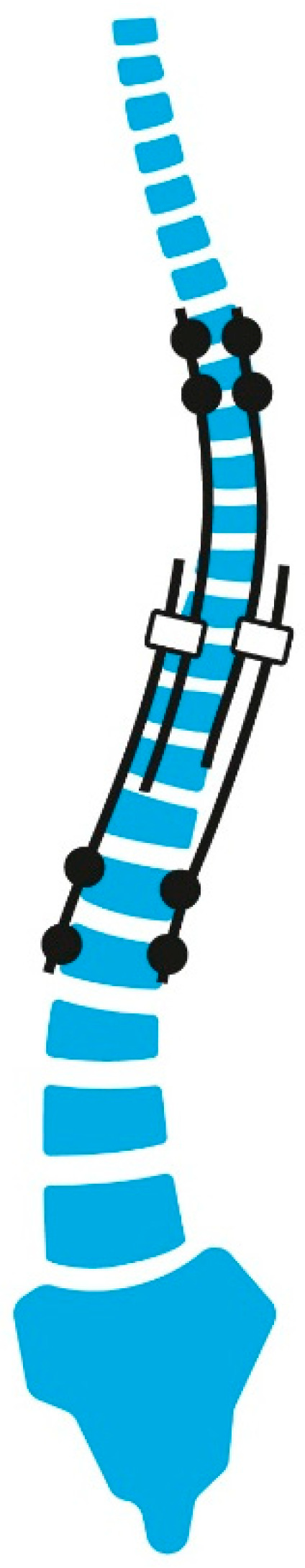
Graphic representation of an example of traditional growing rods (TGR).

**Figure 2:**
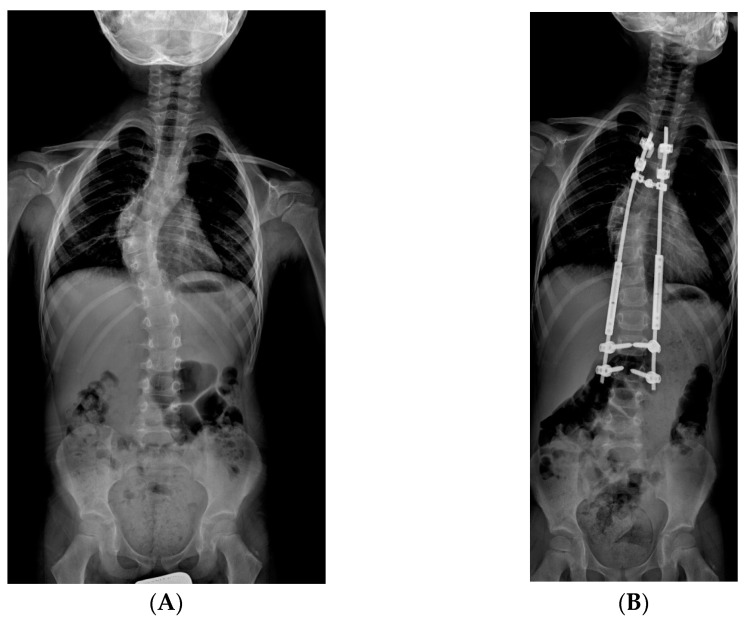

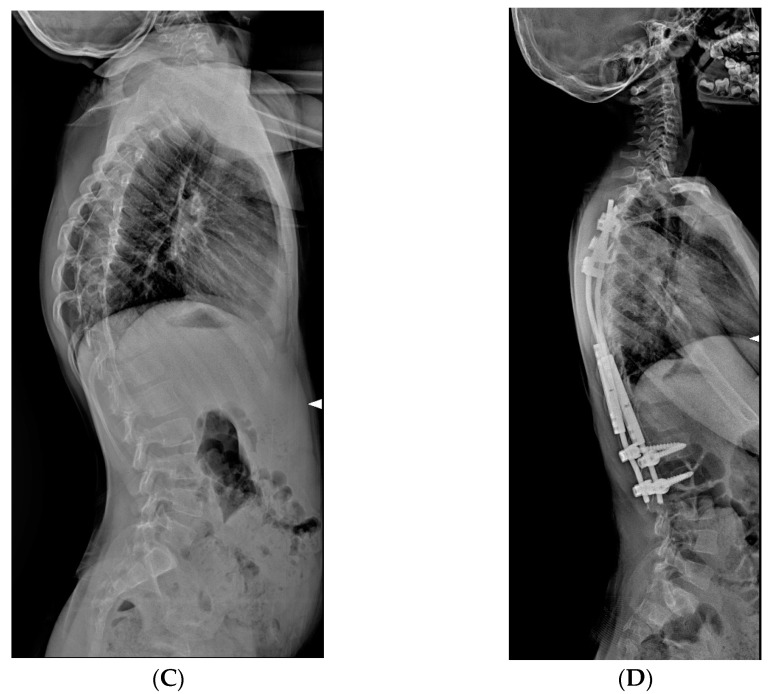
X-rays of early onset scoliosis (EOS) corrected with traditional growing rods (TGR): (**A**) pre-operative antero-posterior X-ray view; (**B**) post-operative antero-posterior X-ray view; (**C**) pre-operative lateral X-ray view; (**D**) post-operative lateral X-ray view.

The principle of TGRs is to anchor the instrumentation to the spine proximally and distally, avoiding exposure of the intervening spinal segment. Growth occurs through this central segment and the deformity is controlled by serial lengthening procedures that allow the spinal growth [16]. The concept of a long subcutaneous rod spanning an unfused spine was first introduced in 1962 by Harrington [17] and thereafter developed by many other authors, especially Morin, who used segmental spinal instrumentation with a “claw” foundation consisting of a downgoing supralaminar or transverse process hook and an upgoing sublaminar hook placed 1 or 2 segments distal [18]. Each rod is composed of two sections, connected by an end-to-end tandem connector through which the lengthenings are performed. The foundations are either pedicle screws or hook “claws” and these anchor points are fused [19]. In neuromuscular patients, the surgeon should consider pelvic fixation for the distal anchor, as Sponseller et al. demonstrated a significantly better correction of pelvic obliquity and coronal deformity using dual rods and iliac screws compared with other types of pelvic fixation [20]. Periodic lengthenings are performed on average every twelve months, and sometimes, in the meanwhile, a thoracic–lumbar–spinal brace is used. Once the child has reached sufficient age and size, a definitive spinal fusion can be performed (Figure 1 and Figure 2).

The TGR technique used in the examined cases consisted of a proximal and distal vertebral anchorage of two rods, each interrupted by a telescopic connector. Lengthening of the posterior structure was performed through a repetitive surgical procedure (on average every 8 months) involving distraction of the rods through the connectors. TGR was both a single rod implant, on the concavity side, and a dual rod implant. The dual rod construct, despite giving more stability, has the limitation of being bulky; for this reason it could not be performed in thin children or those of a very young age. In the TGR construct, both screws and hooks were used for proximal anchorage of the rods; screws were used for the distal one. The initial distraction was of moderate intensity in order to reduce the risk of vertebral anchorage failure and neurological complications.

#### 2.3.2. MCGR: Magnetically Controlled Growing Rods

**Figure 3:**
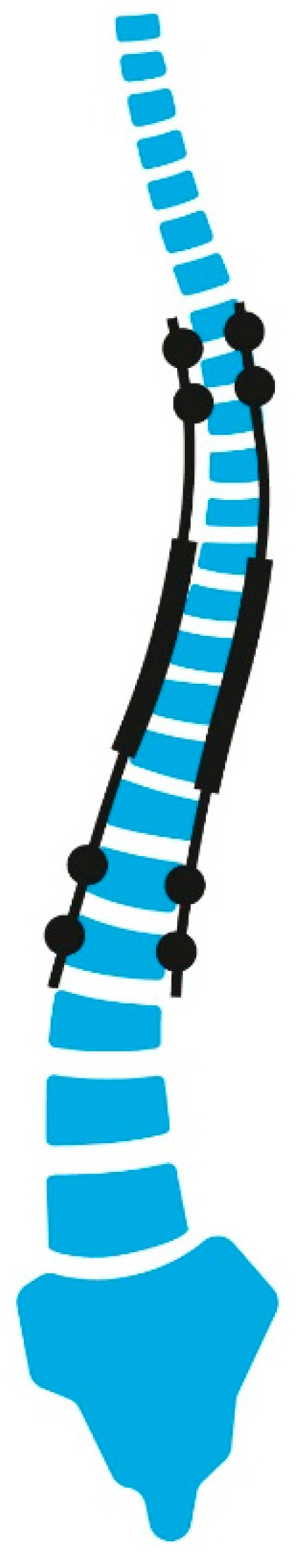
Graphic representation of an example of magnetically controlled growing rods (MCGR).

**Figure 4:**
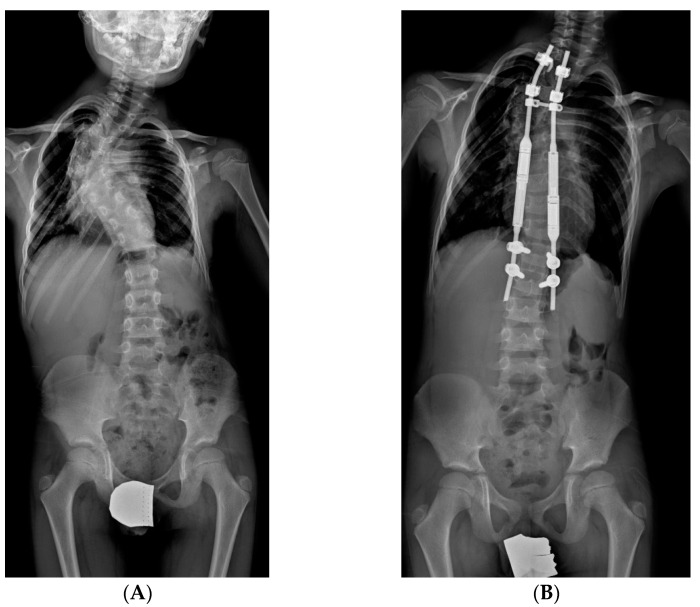

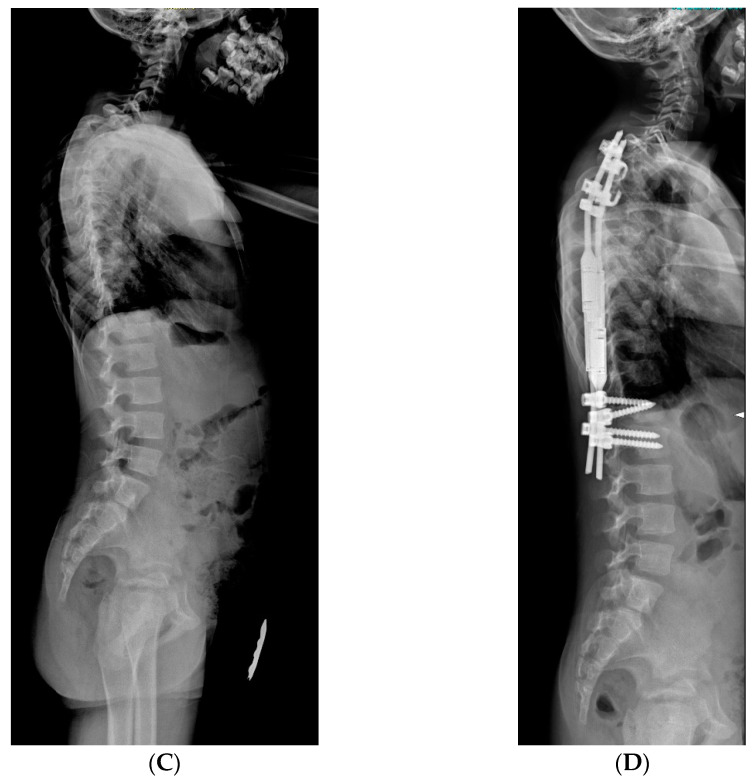
X-Rays of early onset scoliosis (EOS) corrected with magnetically controlled growing rods (MCGR): (**A**) pre-operative antero-posterior X-ray view; (**B**) post-operative antero-posterior X-ray view; (**C**) pre-operative lateral X-ray view; (**D**) post-operative lateral X-ray view.

MCGR are distraction-based systems without the need for repeated surgery; they can allow growth with remote control through an externally placed device to distract the rod via a magnetically driven linear actuator [10,11], on average, every two months. Theoretically, this device can diminish the complications of infection and lack of soft-tissue coverage. It may be especially helpful in children with comorbidities that make the repeated surgeries required by the usual distraction methods particularly difficult (Figure 3 and Figure 4). MCGR involved the same surgical technique as TGR, with the difference that the bar had a magnetic telescopic connector, which was lengthened transcutaneously. Despite the advantage of not requiring multiple surgeries, MCGR systems require more frequent lengthening, approximately every 2 months. We should point out that MCGR corrected partially the sagittal deformity, since the distraction actuator area cannot be contoured as in the TGR construct.

#### 2.3.3. VEPTR: Vertical Expandable Prosthetic Titanium Rib

**Figure 5:**
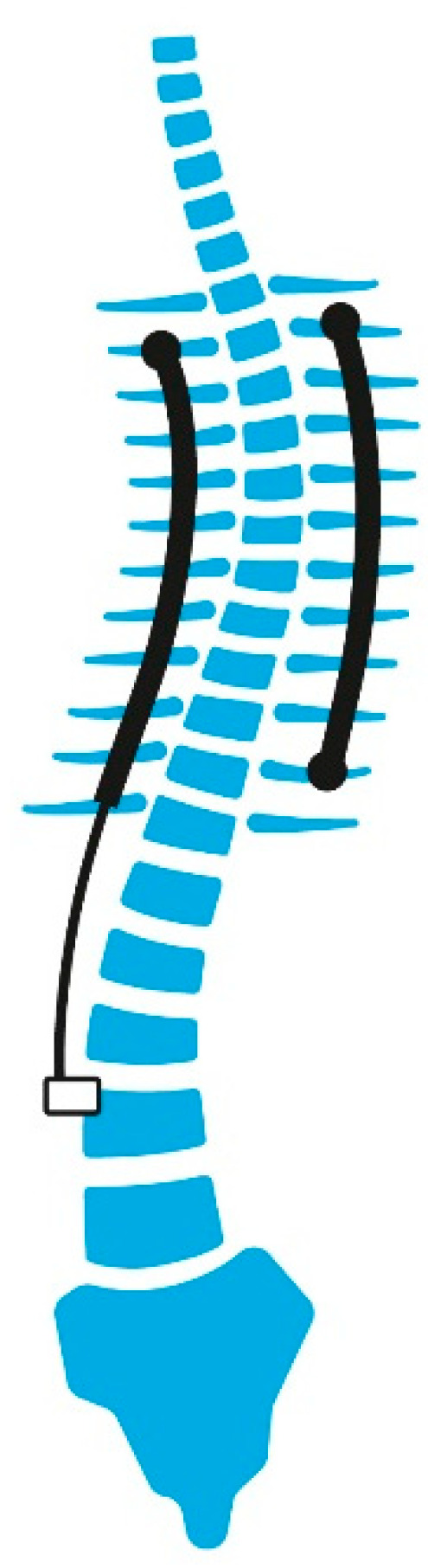
Graphic representation of an example of vertical expandable prosthetic titanium rib (VEPTR).

**Figure 6:**
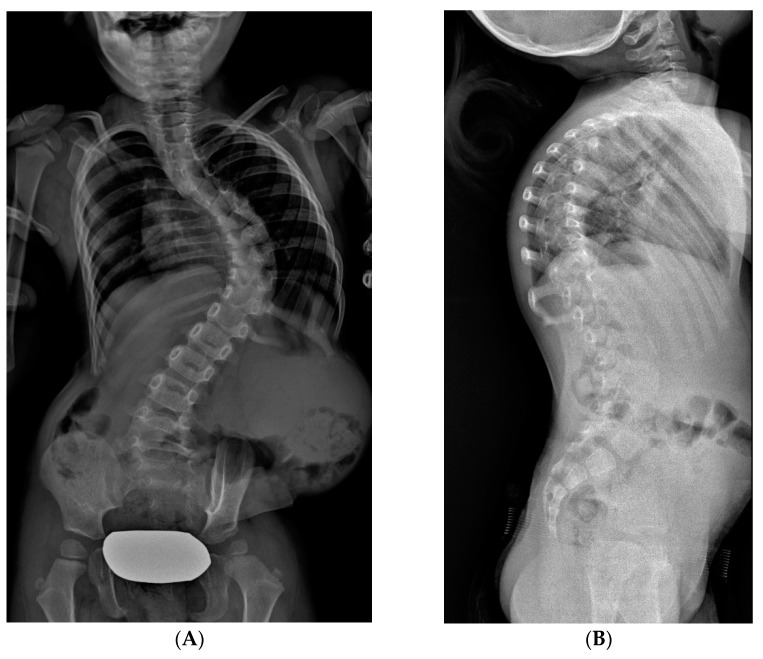

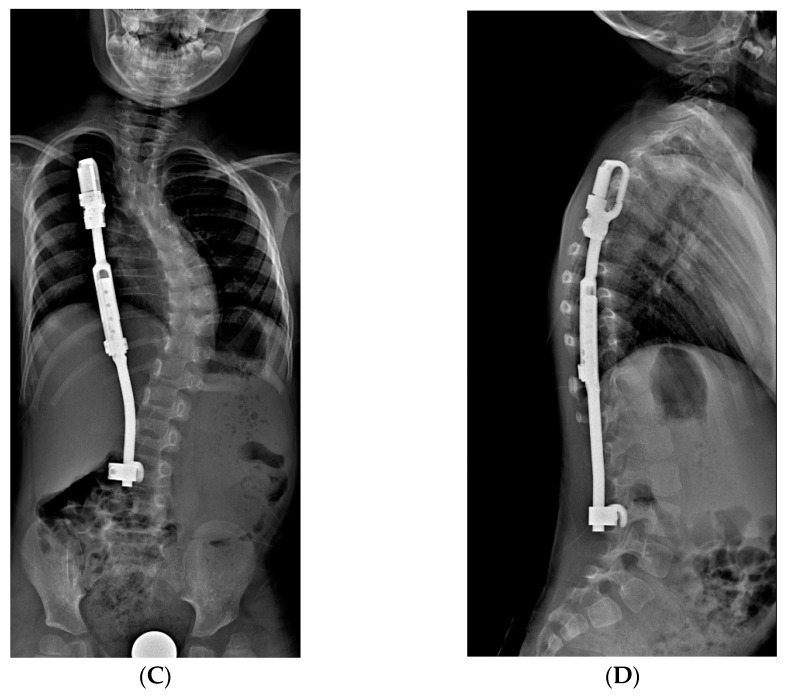
X-rays of early onset scoliosis (EOS) corrected with vertical expandable prosthetic titanium rib (VEPTR): (**A**) pre-operative antero-posterior X-ray view; (**B**) post-operative antero-posterior X-ray view; (**C**) pre-operative lateral X-ray view; (**D**) post-operative lateral X-ray view.

VEPTR is a titanium alloy longitudinal rib distraction device. As with growth rods, repeated lengthenings are required [21]. The goal is to maximize thoracic volume and symmetry of the deformed thorax by lengthening the constricted hemithorax through a transverse opening wedge thoracostomy of the concave side [22], either through osteotomy of fused ribs or intercostal muscle lysis. The scoliosis is corrected indirectly by the thoracostomy, and the thoracic reconstruction is stabilized by the addition of a rib-to-spine or rib-to-pelvis VEPTR construct and another rib-to-rib VEPTR. No bracing is used postoperatively. VEPTRs are lengthened every four to six months. As 50% of the final thoracic volume depends on the growth between the age of 10 to 15 years, the final fusion is preferentially delayed until skeletal maturity (Figure 5 and Figure 6). When spinal deformity is associated with severe thoracic deformity, VEPTR is indicated. The main feature of this technique is that proximally, the bar is anchored to the ribs. Lengthening required surgery room, on average, every 12 months.

### 2.4. Statistical Analysis

All numerical data were expressed in terms of the mean and the standard deviation of the mean; the categorical data were expressed as frequency and percentages. The Shapiro–Wilk test was performed to test normality of continuous variables. The Levene test was used to assess the homoscedasticity of the data. The paired *t* test was performed to assess the differences at different follow-up times. The ANOVA test was performed to assess the differences between 2 groups of numerical, normally distributed, and homoscedastic variables, the Mann–Whitney non parametric test was used otherwise. The ANOVA test, followed by a post hoc Sidak test for post hoc pairwise comparisons, was performed to assess the among groups differences in numerical, normally distributed, and homoscedastic variables. The Kruskal–Wallis nonparametric test, followed by a post hoc Mann–Whitney test with Bonferroni correction for multiple comparisons, was used otherwise. The nonparametric tests were evaluated using the Monte Carlo method to account for the presence of small subgroups. The Spearman rank correlation was used to assess correlations between numerical variables. The Pearson Chi square evaluated using the exact test was performed to investigate relationships between grouping variables. For all tests, *p* < 0.05 was considered significant. All statistical analyses were performed using SPSS v.19.0 (IBM Corp., Armonk, NY, USA).

## 3. Results

### 3.1. Patients’ Treatments

Data from 62 children were analyzed in this study. The population included 39 female and 23 male patients. The mean patient age at first surgery was 7.2 years (range 2.0 to 9.8 years). With regards to the etiology, 26 patients (41.9%) had idiopathic EOS; in 4 patients (6.5%) the etiology was neuromuscular; 16 (25.8%) had syndromic EOS; and 16 (25.8%) had congenital EOS. The mean number of distractions was 3.6 in the TGR group, 15.4 in the MCGR group, and 4.6 in the VEPTR group (*p* = 0.576). The mean interval between distractions was 9.8 months in the TGR group, 3.2 months in the MCGR group, and 7.3 in the VEPTR group (*p* < 0.001). The mean duration of postoperative follow-up after the final operation was 7 years (range 2.0–12.9).

Etiology-based subgroups did not differ by sex, mean age at the initial surgery, mean body mass index (BMI), mean number of lengthening procedures, or mean final follow-up (all *p* < 0.05). Preoperatively, the major curve magnitude had similar Cobb angles regardless of the etiologies.

The three surgical techniques were applied differently according to the etiology (*p* < 0.0005): TGR was the only used approach for neuromuscular EOS (100% of the neuromuscular patients), VEPTR was the most used approach in congenital scoliosis (50% of the congenital scoliosis patients) and syndromic scoliosis (43% of the syndromic scoliosis patients), MCGR was the most used approach in idiopathic scoliosis (74% of the idiopathic scoliosis patients) (Table 1).

The analyzed techniques were not applied differently according to sex and age at first surgery, although there was a tendency for VEPTR to be applied at a younger age (5.9 ± 3.1 years vs. 7.8 ± 1.9 for TGR and 7.4 ± 2.7 for MCGR, respectively, *p* = 0.058).

Spine height correction was 6.8, 9.5, and 10.1 for VEPTR, MCGR, and TGR, respectively, while based on etiology, it was 9.0, 8.3, 10.2, and 6.7, for idiopathic, congenital, syndromic, and neuromuscular, respectively.

### 3.2. Curve Correction

EOS etiologies presented significant differences in the correction of the preoperative major curve, with a lower correction of the coronal Cobb curve in the congenital etiology (19.1° ± 12.7 vs. 30.7° ± 14.3 and 27.4° ± 17.8 for idiopathic and syndromic etiologies, respectively, *p* < 0.0005). The correction of the preoperative major curve was not significantly influenced by age at first surgery and sex.

All techniques led to a statistically significant improvement, with a mean curve reduction of 25.8 95% CI (21.8–29.8) (*p* < 0.0005). Compared with the preoperative measurements, significant differences in curve magnitude correction between subgroups occurred at the final treatment measurements, with MCGR leading to significantly higher correction, both in terms of degrees, as well as percentage of curve correction. The results are reported in detail in Table 2.

### 3.3. Complications

A total of 20 complications were observed. Three cases of surgical site infection (SSI) (one in idiopathic EOS, one in a congenital etiology, and one in a syndromic etiology), one case of proximal junctional kyphosis (PJK) (a congenital EOS), two cases of respiratory complications (both in syndromic etiology), and 14 cases of rod fracture (RF) and/or screw failure were identified (four congenital, six idiopathic, one neuromuscular, and three syndromic). Patients with syndromic EOS and idiopathic EOS had the highest incidence of complications (35%), although this rate was not statistically different from the other groups (25% for congenital and 5% for neuromuscular, respectively). No significant differences were found based on sex, pre-operative Cobb, or age, neither among different techniques.

## 4. Discussion

The main finding of this study is that MCGR presented the highest curve correction among the investigated techniques (Table 1), although the three distraction-based surgical techniques have been applied differently based on the EOS etiology.

The analysis of this case series shows significant differences in curve magnitude correction between treatment subgroups occurred at the final measurements, when patients with MCGRs had significantly larger correction 33.95° (COBB Δ65.0–31.1) than VEPTR 19.78° ((COBB Δ68.6–48.8) reduction of mean Cobb angle, respectively), and TGRs (correction of 21.76°). Tahir M. et al. analyzed the radiological outcomes of patients with EOS who had undergone spinal fusion after distraction-based spinal growth modulation using either TGRs or MCGRs and found that the mean Cobb angle improved by 25.1° in the MCGR group and 23.2° in TGR group [23].

Few studies analyzed the results of the growing rod approach according to the etiologic subtype of EOS, and these studies allowed us to further analyze the results based on the etiology [5,6,7,8,9,10,11,12,13,14,15,16,17,18,19,20]. The mean preoperative Cobb angle of the major curve was 64.8 degrees in this study and a significant improvement in curve magnitude was achieved in terms of magnitude curve improvement with 25.8° Cobb correction. However, results differed based on the etiology: Idiopathic EOS presented a 46.5% reduction of the major curve, while 31.1% of correction was achieved for congenital EOS, syndromic was 44.8%, while neuromuscular had no significant variation. With respect to congenital anomalies, these results were directly compared with the previously reported findings of Elsebai et al. [24], who demonstrated that the percentage of major curve correction from preoperative to postoperative initial was 31% and from preoperatively to the latest follow-up was 29%.

Treatment of EOS is still characterized by a high rate of complication [25]. The high incidence of complications is the result of a long treatment period due to the young age of patients at first surgery and the high number of surgical procedures required. Regardless of the different results based on EOS etiology and specific growing rod treatment, no differences were found in terms of complications (*p* = 0.830 for treatment and *p* = 0.721 for etiology). This study included a large sample of EOS treated with GR systems according to the different etiologies in a single center, reducing the variability in the indication for surgery, timing of surgery, postoperative care, and complication management. Overall, patients of this series had a rate of 32% of complications, and 16 of them needed unplanned surgery to manage it. These results are in contrast with the study conducted by Teoh et al. [26], who reported higher rates of complication (70% of complications in the MCGR group and 77% in the TGR group). Our results also consisted of a lower number compared to the results reported by Bess et al. in 2010, who found 57% complications in 140 participants [27]. In contrast to a defined fusion method, the high number of implant-related complications is to be referred to the dynamic nature of the treatment and to the periodic pressures applied to the rods [28].

One of the most common mechanical complications is rod fracture (RF), which can have a variable impact on the course of treatment, requiring a possible unplanned surgery. We had 14 cases of RF, and we found no significant differences among pre-operative major curve Cobb. This result is consistent with the study conducted by Hosseini et al., who demonstrated that RF and NRF patients had statistically similar mean pre-op major curve size and max kyphosis (*p* = 0.279; *p* = 0.619, respectively) [29]. PJK is another frequent complication of EOS treatment. In our study, only one case of PJK was seen. Zarei M. et al. studied the results and complications of all-pedicle screw dual GR instrumentation in the treatment of EOS and encountered three cases (7%) of PJK during the study period, none of which required revision surgery. According to their study, pre-operative TK was the only significant risk factor for the development of PJK [30]. Three cases of SSI were observed in our population. The whole patients were treated appropriately with antibiotic therapy, and no additional surgery was required. Our results are similar to Liang J. et al., who observed only four cases of infections (11%), of which two were SSI. They demonstrated that the duration between GR lengthening procedures (OR: 1.121; *p* = 0.003) and duration of follow-up (OR: 1.079; *p* = 0.001) maintained its significance in predicting likelihood of postoperative complications [31].The infection rate in our population was much lower compared to the 6.7% reported by Mackenzie (4% vs. 6.7%) in 201335; this is consistent with the knowledge that the frequency of infections increases as more surgical procedures are performed [32]. These findings confirm what is in the literature that high prevalence of complications during treatment and follow-up correlates to the duration of growing rod treatment. The number and frequency of distractions are other factors that can influence the infection rate [33].

This study presents some limitations: syndromic and congenital EOS subtypes encompass a wide range of diagnosis with different medical comorbidities associated that predispose patients to different types of complications. While the aim of this study was to compare, in a large series of patients, the potential and limitations of the different distraction-based techniques to establish the most suitable surgical approach for patients affected by EOS, to properly achieve the objectives, the corrective procedures performed on EOS patients should be conducted within the same group of patients. Unfortunately, this is a challenging and rare condition that does not favor such study design, as confirmed by the current lack of high-level studies, and future studies should aim at increasing the study level with randomized controlled trials to investigate this topic. Furthermore, this work is also limited by its consideration of a retrospective design. Finally, while commonly indicated for >40 degrees, some patients underwent surgery also for lower Cobb values in cases of rapidly progressing deformities. Despite these limitations, this study provides a large series of patients treated in the same institution by the same specialized team and provides insights into the different approaches with respect to indication, results, and complications, for the management of EOS.

## 5. Conclusions

Different growing-rod techniques are applied based on EOS etiology. While all EOS etiologies benefited from this surgical approach, congenital EOS had poorer results. Overall, MCGR has been the preferred option for idiopathic EOS and appears to be the most effective in correcting the primary curve.

## Figures and Tables

**Table 1:** Growing-rod techniques according to etiology.

	TGR	VEPTR	MCGR
N	16%	0%	0%
I	32%	7%	74%
C	24%	50%	13%
S	28%	43%	13%

C: congenital, I: idiopathic, N: neuromuscular, S: syndromic. MCGR: magnetically controlled growing rod; TGR: traditional growing rods; VEPTR: vertical expandable prosthetic titanium rib.

**Table 2:** Mann–Whitney post hoc test with Bonferroni correction for multiple comparisons. VEPTR: vertical expandable prosthetic titanium rib; MCGR: magnetically controlled growing rod; TGR: traditional growing rods.

	VEPTR	MCGR	TGR	TOTAL	Kruskal–Wallis Test	Post Hoc Mann–Whitney Test
N	14	23	25	62		
Cobb pre Mean (SD)	68.6 (19.9)	65.0 (15.8)	62.4 (23.5)	64.8 (19.9)	*p* = 0.426	-
Cobb post Mean (SD)	48.8 (16.0)	31.1 (18.0)	40.7 (18.9)	38.9 (18.9)	*p* = 0.008	VEPTR vs. MCGR *p* = 0.012 VEPTR vs. TGR *p* = 0.421 MCGR vs. TGR *p* = 0.219
% curve correction Mean (SD)	27.2 (19.1)	53.3 (20.8)	34.8 (20.0)	39.9 (22.5)	*p* = 0.001	VEPTR vs. MCGR *p* = 0.002 VEPTR vs. TGR *p* = 0.572 MCGR vs. TGR *p* = 0.009
Cobb correction Mean (SD)	19.7 (16.7)	33.9 (14.3)	21.7 (13.6)	25.8 (15.7)	*p* = 0.005	VEPTR vs. MCGR *p* = 0.042 VEPTR vs. TGR *p* = 0.976 MCGR vs. TGR *p* = 0.012

## Data Availability

The raw data supporting the conclusions of this article will be made available by the authors on request.
